# Resolutions of Respect

**Published:** 1874-06

**Authors:** 


### RESOLUTIONS OF RESPECT.

At a meeting of the dentists of this city, yesterday, to pass resolutions concerning the death of Dr. D. C. May, Dr. J. Taft was elected Chairman, Dr. H. M. Reid, Secretary, and Drs. Meredith and Osmond were appointed as the committee to draw up resolutions expressive of the feeling of the meeting.

The following were offered and approved:

"WHEREAS. An all-wise Providence, whose ways are unsearchable and past finding out, has seen fit to remove from our midst, almost in the beginning of his professional career, one of our number, D. C. May.

"Resolved. That while we bow in humble submission to the will of Him who doeth all things well, we can not refrain from expressing our sorrow at the death of one of the members of our profession.

"Resolved. That we tender our heartfelt sympathies to the family of the deceased in this their sudden bereavement.

"Resolved. That a copy of these resolutions be sent to each of the daily papers, and also to the family of the deceased."

Cincinnati, May 20th 1874.

